# User tendency-based rating scaling in online trading networks

**DOI:** 10.1371/journal.pone.0297903

**Published:** 2024-04-16

**Authors:** Soohwan Jeong, Jeongseon Kim, Byung Suk Lee, Sungsu Lim

**Affiliations:** 1 Department of Computer Science and Engineering, Chungnam National University, Daejeon, Republic of Korea; 2 Department of Computer Science, University of Vermont, Burlington, VT, United States of America; Chunghwa Telecom Co. Ltd., TAIWAN

## Abstract

Social networks often involve the users rating each other based on their beliefs, abilities, and other characteristics. This is particularly common in e-commerce platforms where buyers rate sellers based on their trustworthiness. However, the rating tends to vary between users due to differences in their individual scoring criteria. For example, in a transaction network, a positive user may give a high rating unless the transaction was unsatisfactory while a neutral user may give a mid-rating, consequently giving the same numeric score to different levels of satisfaction. In this paper, we propose a novel method called *user tendency-based rating scaling*, which adjusts the current rating (its score) based on the pattern of past ratings. We investigate whether this rating scaling method can classify between “good users” and “bad users” in online trade social networks better when compared with using the original rating scores without scaling. Classifying between good users and bad users is especially important for *anonymous* rating networks like Bitcoin transaction networks, where users’ reputations must be recorded to preclude fraudulent and risky users. We evaluate the proposed rating scaling method by performing user classification, link prediction, and clustering tasks, using three real-world online rating network datasets. We use both the original ratings and the scaled ratings as weights of graphs and use a weighted graph embedding method to find node representations that reflect users’ positive and negative information. The experimental results showed that using the proposed rating scaling method outperformed using the original (*i.e*., unscaled) ratings by up to 17% in classification accuracy, and by up to 2.5% in link prediction based on the AUC ROC measure, and by up to 21% in the clustering tasks based on the Dunn-index.

## Introduction

Social networks provide a platform for users to interact with each other by exchanging ratings, positive or negative. Analyzing the behavioral patterns of users concerning the ratings in such networks is a fundamental task of importance. For example, in a cryptocurrency exchange like Bitcoin, buyers rate sellers based on the level of trust they have in them after a completed transaction. Similarly, in the Wikipedia network, administrators rate the general editors based on their level of support or opposition. Researchers have conducted studies to identify meaningful content on rating networks. Kumar et al. [1, 2] proposed fairness and goodness of node. Fairness is an index that indicates how fairly the scoring user gives the rating, and goodness is an index that indicates how good the person receiving the rating is. By using fairness and goodness indices, they were able to predict the weight (rating) of edges and identify fraudulent users who assign unfair ratings. Pareja et al. proposed EvolveGCN [3], which learns node embedding over time by combining dynamic graph with graph convolutional network (GCN) [4] and sequence embedding techniques. They used a weighted graph to classify edges as positive or negative and verified the representation of node embedding in a time series graph.

**Fig 1:**
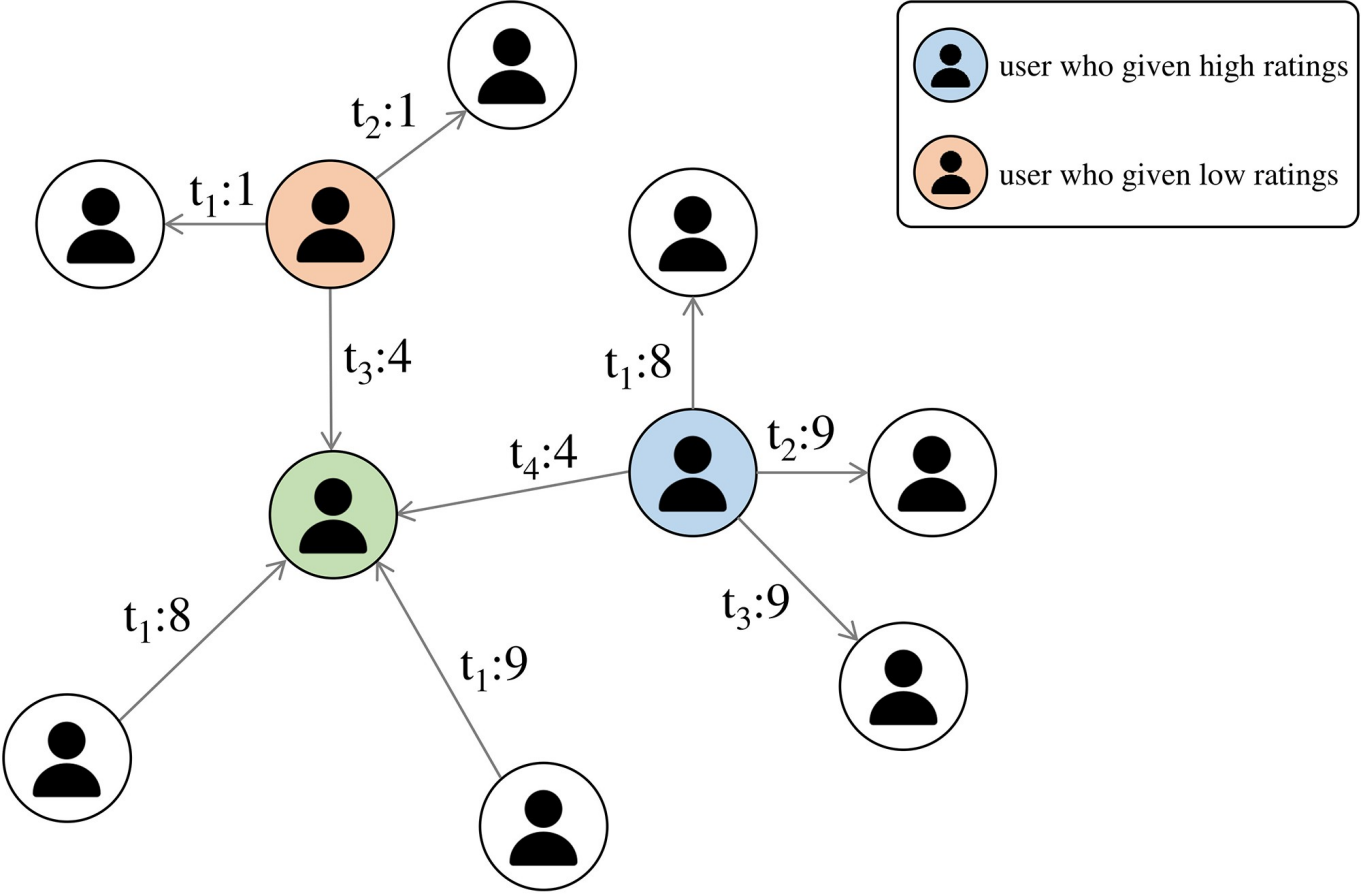
Example of rating network. Orange users usually tend to give low scores. Conversely, blue users usually tend to give high scores. Could it be said that the 4 points each user gave to the green user are semantically the same? 4 points given by the orange user should be weighted positively, and the 4 points given by the blue user should be weighted negatively.

However, previous studies that utilized edge weights in rating networks did not take into consideration the subjective nature of ratings. Each user has their own subjective tendency to rate others based on their own criteria such as Fig 1. For example, in a trading network, when the rating ranges between −10 (completely negative) and + 10 (completely positive), and the satisfaction of the transaction is normal, someone will give 0 points, which is the median in the rating range. On the other hand, a person with a positive subjective tendency may give a non-zero positive rating even if their satisfaction with the transaction is moderate. In other words, even with the same rating, it may reflect different levels of satisfaction based on the user’s subjectivity. Therefore, the objective of this paper is to introduce a *user’s tendency-based scaled rating* that takes into account the user’s subjectivity.

In defining the scaling method, we consider two crucial factors as follows. Firstly, we adjust the current rating $r_{u}^{\left(n\right)}$ given at time *n* to a scaled rating $s_{u}^{\left(n\right)}$ based on their past ratings $\left\{r_{u}^{\left(0\right)},r_{u}^{\left(1\right)},\ldots ,r_{u}^{\left(n-1\right)}\right\}$ to reflect the user’s level of satisfaction compared to their past rating history. Secondly, we assigned different weights to past ratings that were relatively distant (*e.g*., $r_{u}^{\left(0\right)}$) and recent past ratings (*e.g*., $r_{u}^{\left(n-1\right)}$) to give more importance to the user’s recent rating tendencies, as these tendencies may change over time.

To evaluate the efficacy of the scaling method, we conducted weighted graph embedding learning on three real-world rating networks: Bitcoin-Alpha, Bitcoin-OTC, and Wiki-RfA. We used the ratings before adjustment (original ratings) and after adjustment (scaled ratings) as weights in embedding learning. We obtained the node embeddings of each version by applying both original and scaled ratings to a model that combined weighted node2vec [5] and graph auto-encoder (GAE) [6]. Since the weight cannot be a negative value due to the characteristics of weighted node2vec, the absolute value of the rating was used in this work. Node embeddings obtained through weighted node2vec reflected the degree of positive and negative meaning. We defined these embeddings as features of each node and applied them to GAE to obtain node embeddings that reflect positive and negative information.

Subsequently, we performed experiments to verify whether the rating adjustment could enhance the classification of good and bad users, which corresponds to the purpose of this paper. In order to verify not only the classification performance, but also the power of the embedding representation, we additionally performed link prediction and clustering, and compared the corresponding performances. The results showed that the embedding using scaled ratings had better node representation than original ratings, leading to superior classification performance for good users and bad users.

The following sections in this paper consist of **Datasets**, **Method**, and **Experiments**. In the **Datasets** section, we introduce real-world datasets utilized in our experiments, while in the **Method** section, we describe the proposed rating scaling algorithm and the graph embedding method. Finally, in the **Experiments** section, we demonstrate three tasks and discuss their performance evaluation, including the results of applying the rating scaling method.

## Related works

### User classification

Due to the advancement of the Internet and e-commerce, rating networks involving the exchange of ratings within interactions between user products can be readily observed. Studies that aim to identify significant users within the network or to detect fraud users through the analysis of users in such rating networks have been consistently proposed. The studies have been extensively conducted to analyze timestamps and text reviews left by users regarding products in order to identify malicious users or fraudulent and deceptive products [7–10]. Li et al. proposed a reputation-based algorithm that involves the aggregated difference between a user’s rating and the ranking of the corresponding object (*i.e*., product) [11]. Recently, with the continuous advancement of graph neural networks (GNN), Zhang et al. utilized the GNN-based user representation learning framework, GraphRfi, to identify fraudulent users and contribute to enhancing the performance of recommendation systems [12].

Previously, obtaining the rating relationships between users was difficult, so information between users and products was utilized. However, recent studies have introduced approaches that utilize rating information among users, resolving user analysis and prediction problems within user-user networks. Mishra et al. proposed an algorithm for calculating user bias and prestige based on the idea that the bias of each user reflects their tendency to trust or distrust neighboring users, which is closely related to credibility. They found that heavily biased user recommendations are undesirable [13]. Furthermore, Kumar et al. defined goodness and fairness for each individual user and proposed an algorithm that predicts the weights of edges (*i.e*., ratings) based on the scores of each user within the network [1, 2]. The previous studies conducted modeling through users’ biases or deviations, defining scores for each user to predict anomalies. On the contrary, this study proposes scaling ratings based on the history of individual users’ ratings. Subsequently, it utilizes adjusted ratings to conduct node representation learning based on GNN, aiming to find explicit node representations for various sub-tasks, including user classification.

### Graph embedding methods

Graphs representing objects (nodes) and relationships (edges) between objects are ubiquitous (*e.g*., social networks, chemical compound structures, citation networks, transaction networks, etc.). The development of graph representation learning, which projects high-dimensional graph nodes or links into low-dimensional vectors, has made solving downstream tasks that utilize graph structures more feasible [14–19]. Methods for obtaining vector representations of nodes can be broadly categorized into three approaches: (a) matrix factorization-based methods, (b) random walk-based methods, and (c) deep learning-based methods.

(a) **Matrix factorization-based methods**: GraRep [20] is a matrix factorization-based graph embedding method that decomposes higher-order adjacency matrices to capture node similarity, preserving global structural information across various scales. Likewise, HOPE [21] is a matrix factorization-based methodology, aiming to capture asymmetric transitive relationships in networks by preserving high-order proximity information.

(b) **Random walk-based methods**: random walk-based graph embedding methods treat sequences of nodes generated from a starting source node on the graph as sequences of words and employ language modeling methods (*e.g*., skip-gram) to learn node representations. Notably, there are DeepWalk [22] and node2vec [5], with DeepWalk learning structural regularity, and node2vec enhancing this by incorporating in-out and return parameters to balance breadth-first and depth-first exploration.

(c) **Deep Neural Network-based methods**: while the previously mentioned methodologies solely utilized the structural information of graphs, the utilization of deep learning-based GNN has made it possible to incorporate node feature information as well. As a prominent example, LINE [23] preserves both local and global network structures through first-order and second-order proximities, and learns node representations. GCN [4] can encode both the structural information of the graph and node features. GraphSAGE [24] learns node representations via sampling and aggregation features within the local neighborhood of each node in the graph. In this study, we used node2vec and a deep learning-based GAE using GCN as an encoder to perform embedding for graphs with weights and direction. Details on this are described in Section **Graph embedding method**.

## Materials and methods

### Datasets

**Fig 2:**
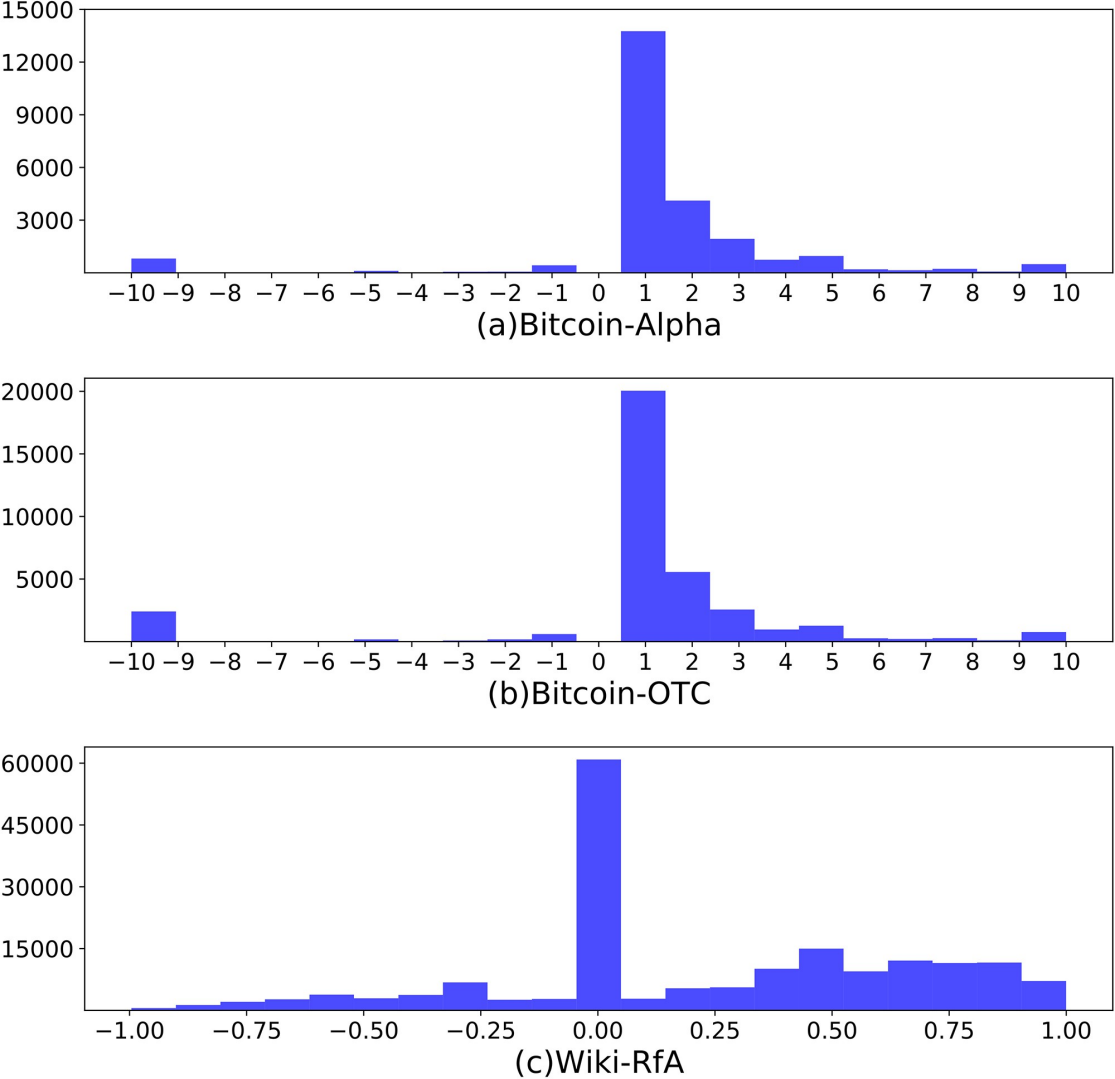
The histogram of rating distributions for three datasets. The x-axis represents the rating value of each dataset, and the y-axis represents the number of ratings. (a) Bitcoin-Alpha and (b) Bitcoin-OTC range between −10 and + 10 except for 0, with most ratings from +1. Since (c) Wiki-RfA was created based on sentiment scores, it shows a continuous distribution contrary to Bitcoin data.

In this section, we present the proposed scaling algorithm and the three real-world rating network datasets to which we applied it. Additionally, we demonstrate the effects of the scaling algorithm through illustrative examples. Please refer to Fig 2 in which an example is shown.

#### Bitcoin-Alpha and Bitcoin-OTC

First, we used two cryptocurrency exchange datasets: the Bitcoin-Alpha dataset and the Bitcoin-OTC dataset [1, 2]. Alpha is a who-trust-whom network of people who traded using Bitcoin on the Bitcoin-Alpha platform from November 2010 to January 2016. Due to the nature of cryptocurrency, users are guaranteed anonymity, so recording users’ reputation information is an indispensable problem to prevent fraudulent and risky users. The dataset consists of 3,783 users (nodes) and 24,186 tradings (weighted directed edges) between users. Each user could be a buyer (rater) and a seller (ratee) simultaneously. The ratings range from −10 to + 10 and the negative ratio is about 7%.

OTC is also a who-trust-whom network, collected on the Bitcoin-OTC platform during the same period as Alpha. The dataset consists of 5,881 users and 35,592 trades between users, where the ratings range from −10 to + 10 like Alpha, and the negative ratio is about 11%.

#### Wiki-RfA

The Wiki-RfA dataset [25] is designed to enable Wikipedia editors to apply for administrator positions by having other members of the community vote in support (+ 1), neutral (0), or opposition (−1) of the candidate. Each vote includes a text comment and a single discrete opinion. To ensure a diverse rating distribution during preprocessing, we utilized VADER [26] to conduct sentiment analysis on the text comments and used the sentiment score as the rating between the two users. The sentiment score-based rating ranges from −1 to + 1. The dataset includes 10,835 users and 159,388 ratings based on sentiment scores, with a negative ratio of approximately 16%.

### Proposed method

#### Scaling algorithm

In this section, the proposed scaling algorithm is demonstrated with some equations. Our proposed algorithm is simple but effective.

Assuming that a user *u* gives a rating $r_{u\to v}^{\left(n\right)}$ to another user *v* at the time *n*, $r_{u\to v}^{\left(n\right)}$ is adjusted a scaled rating $s_{u\to v}^{\left(n\right)}$ as in Eq 1. In this case, *μ*_*u*_ represents the average of the ratings ($\left\{r_{u}^{\left(0\right)},r_{u}^{\left(1\right)},\ldots ,r_{u}^{\left(n-1\right)}\right\}$) that user *u* has given to other users before time *n*. The parameter *θ* is a scale parameter that allows you to adjust how much of the history of past ratings is reflected in the current rating. As the value of *θ* increases, the current rating is more heavily reflected, and as the value of *θ* decreases, the influence of past rating history becomes more significant.

$$\begin{array}{c} \begin{array}{c} Scaled\;rating\;\;s_{u\to v}^{\left(n\right)}=\frac{r_{u\to v}^{\left(n\right)}-\mu_{u}}{\theta }+r_{u\to v}^{\left(n\right)} \end{array} \end{array}$$ (1)

The parameter *μ*_*u*_ shown in Eq 1 means the average of the ratings given by user *u* in the past. To account for time-decay in this equation, *i.e*., to assign different weights *w* based on the time point when user *u* gave a rating (distant past vs. near past), *μ*_*u*_ is formulated as shown in Eq 2.

$$\begin{array}{c} \begin{array}{c} \mu_{u}=\frac{\sum_{k=0}^{\beta }w^{k}\mu_{k}}{\sum_{k=0}^{\beta }w^{k}},\;\;\;\;\left(0<w<1\right) \end{array} \end{array}$$ (2)

$$\begin{array}{c} \begin{array}{c} \mu_{k}=\sum_{i\in R_{k}}\frac{r_{i}}{|R_{k}|}. \end{array} \end{array}$$ (3)

**Fig 3:**
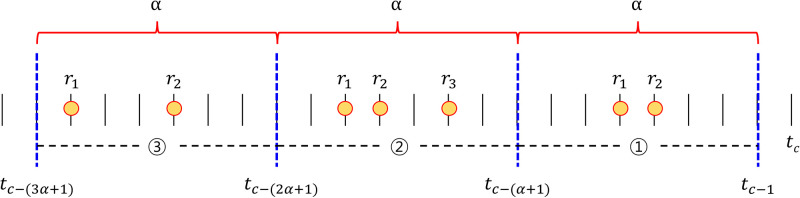
Parameters for time-decay function. The parameter *α* means an arbitrary period, and *t*_*c*_ means the current point in time. Here, “arbitrary” can be any period, such as 1 week (7 days), 1 month (30 days), 3 months (90 days), etc.

The parameter *k* in Eq 2 represents the *k*-th interval among arbitrary past intervals. The parameter *β* represents the time period containing the time when user *u* made the first transaction. In other words, a larger *β* indicates that the user has had a transaction history for a longer period. Referring to Fig 3, the interval from time *t*_*c*−(*α*+1)_ up to just before the current point in time *t*_*c*_ can be defined as the *k* = 1 interval. Let *T*_*k*_ be denoted as *T*_*k*_ = [*t*_*c*−(*k*+1)*α*−1_, *t*_*c*−*kα*−1_) where, *k* = (0, 1, 2, …), and *R*_*k*_ be the set of ratings existing in *T*_*k*_. If *R*_*k*_ = {*r*_1_, *r*_2_, …, *r*_*N*_}, the average *μ*_*k*_ is expressed as Eq 3, where |*R*_*k*_| is the number of ratings in the interval *T*_*k*_. We extensively discuss the configuration and analysis of the hyper-parameters corresponding to *w* of Eq 2 and *α* of Fig 3 in the **Parameter setup** in the **Experiments** section.

In other words, after calculating the average for each interval, the time decay function is designed by performing a weighted average operation through the weight corresponding to the square of *w* for each average. The parameter *α* in Fig 3 represents an *arbitrary* time period (*e.g*., a week, a month, 6 months, etc.) Ratings given within the same unit period are adjusted with the same time-decay weight, denoted by *w* (0 ≤ *w* ≤ 1). As *w* increases (closer to 1), it reflects ratings from the distant past more heavily, and as *w* decreases (closer to 0), it reflects ratings from the recent past more significantly.

**Fig 4:**
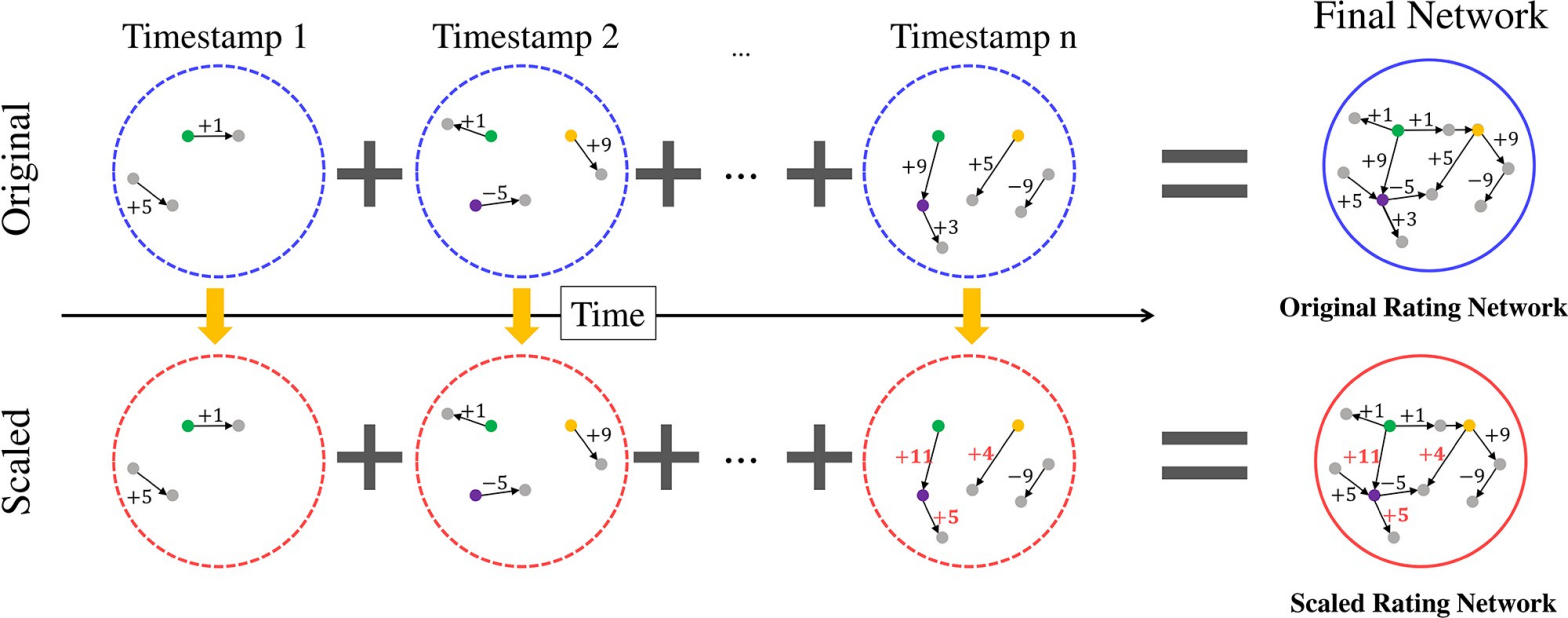
The process for structuring rating networks by scaling rating over time. Considering the ratings given in the past by each user, a scaled rating network is constructed in which the ratings are adjusted every timestamp and the finally adjusted ratings are regarded as the weight of each edge.

Fig 4 illustrates that the ratings given at each timestamp are adjusted based on the ratings given by each user in the past using the proposed scaling algorithm. For instance, the green user gave +1 on both timestamps 1 and 2. Assuming no special transactions occurred from timestamp 3 to *n* − 1, if a +9 point was given to another user at timestamp *n*, the scaling algorithm adjusts this point to +11. Similarly, ratings given by purple and orange users at timestamp *n* are adjusted based on their ratings before *n*. Finally, the adjusted ratings are aggregated at each timestamp to construct a scaled rating network. We apply the original and scaled rating networks to a graph embedding method to determine which version provides more explicit node representation and better classification of good and bad users. Therefore, in the next section, the graph embedding methods adopted in this work are described.

#### Graph embedding method

We performed directed and weighted graph embedding to demonstrate the effectiveness of the scaling algorithm. As mentioned, we utilized weighted node2vec [5] and GAE [6] as graph embedding methods. node2vec is a well-known algorithm based on random walk paths on graphs. node2vec is a methodology in which several parameters are added to DeepWalk [22]. DeepWalk was proposed by referring to skip-gram used in word2vec [27] in the field of natural language processing. Skip-gram was trained to predict words in the neighbors of a specific word in a sentence. That is, words that are similarly used in sentences are close to each other even after word embedding learning.

If this concept is applied to graph networks, nodes with similar characteristics will exist in similar positions in the embedding space even after graph embedding learning. node2vec can control whether Breadth-First Search (BFS) or Depth-First Search (DFS) is performed when extracting a node sequence by adding parameters *p* and *q* to DeepWalk. The parameter *p* is a return parameter, and as the value increases, the probability of moving to a new node increases. That is, the probability of revisiting a previously visited node is reduced. Conversely, as *p* becomes smaller, it revisits previously visited nodes and hovers around there. The parameter *q* is an in-out parameter. As the value of *q* increases, the probability of visiting a region increases, and as the value decreases, the probability of visiting a new node increases. Since *p* and *q* represent opposite properties, it is important to find the optimal values according to the target. Finally, the weighted version of node2vec reflects the weight of the edge connected between two nodes.

Assuming that the set of weights between neighboring nodes extending from a specific node *a* is $a_{nbrs}^{out}=\left\{a_{nbr1}^{out},a_{nbr2}^{out},\ldots ,a_{nbrN}^{out}\right\}$, the weight between node *b* among the neighboring nodes of *a* is $a_{b}^{out}$ Let’s assume when extracting a node sequence, the probability of moving from a to b is as shown in Eq 4.

$$\begin{array}{c} \begin{array}{c} Probability\;\left(a\to b\right)=\frac{a_{b}^{out}}{\sum_{i\in a_{nbrs}^{out}a_{i}^{out}}} \end{array} \end{array}$$ (4)

However, in rating networks, it is common for the range of weights to show negative values. Therefore, in this work, in order to apply weighted node2vec [5], the rating range was modified to have a value greater than 0. At this time, if the range of rating is simply moved in parallel, since the smallest rating value becomes 0, the probability of moving through the edge converges to 0, which is affected when extracting a sequence. To avoid this phenomenon, we took an absolute value for the rating value, which can reflect the semantic magnitude relationship of how good and how bad rather than reflecting the meaning of good and bad. That is, for example, assuming a rating network in which the range of rating values is [−10, +10], giving −10 or +10 points could be said to be actively expressed, and conversely giving −1 or +1 point could be said to be a passive expression of intention.

**Fig 5:**
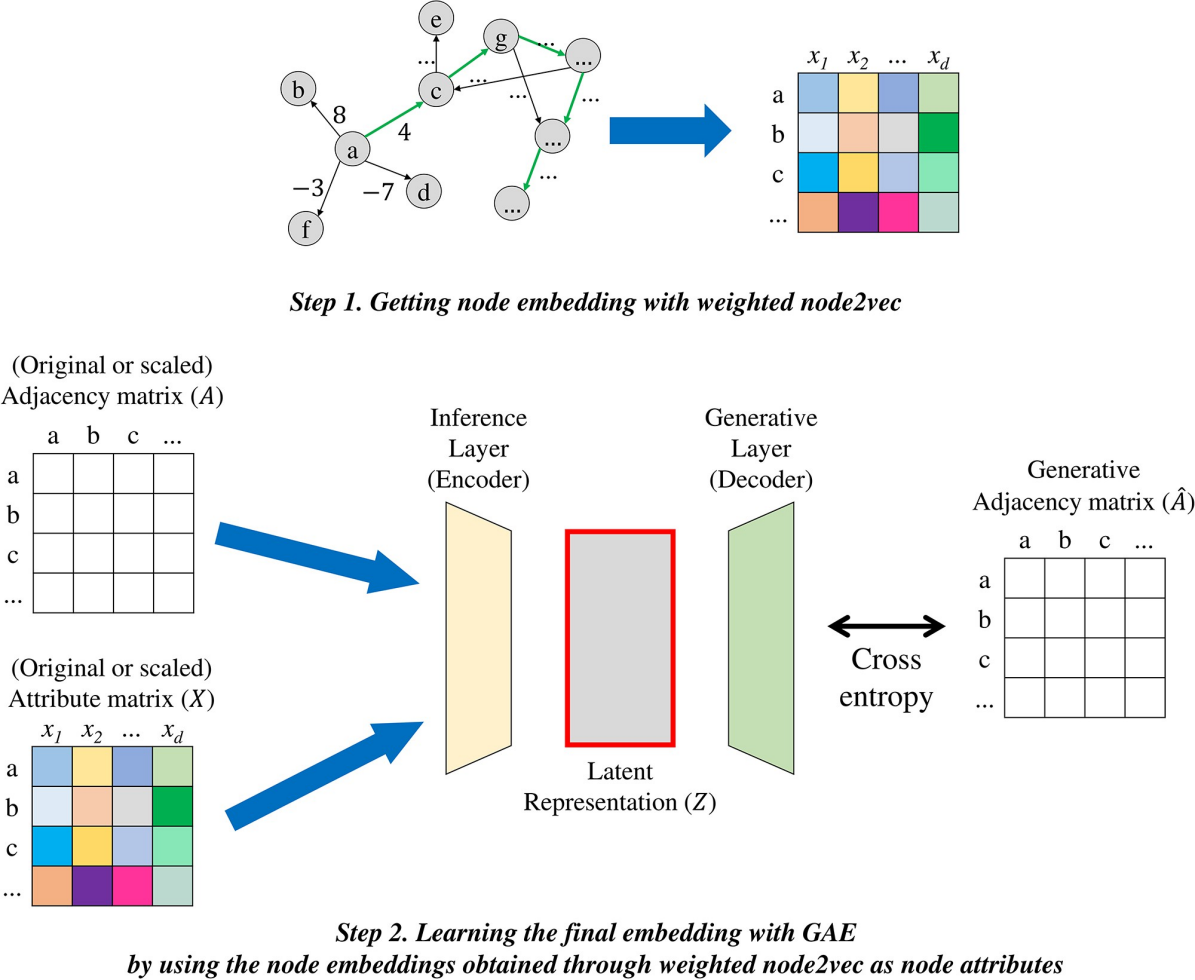
Process of graph embedding. We proceeded with graph embedding in two stages. In *step 1*, the embedding of the node is obtained using the weighted node2vec method. In *step 2*, final graph embedding is conducted by using the node embedding obtained in *step 1* as an feature of each node and feeding it into the GAE embedding model.

As shown in Fig 5, each node representation reflecting the magnitude of ratings obtained by weighted node2vec [5] was used as node features and applied to the GAE [6] model to learn the final node representation. GAE is a popular graph embedding model based on GNN. As shown in *Step 2* of Fig 5, the GAE module includes an encoder and a decoder. The encoder consists of GCN layers, and the decoder consists of an inner-product layer. In other words, GCN is used to encode each node in the graph network into the latent feature space, and the graph is reconstructed using the inner-product of the expression of the latent space. The reconstruction error is minimized and node embedding is learned. In *Step 1* of Fig 5, the positive and negative degrees were reflected, and in *Step 2*, the actual rating value was reflected. The GAE takes the adjacency matrix (*A*) and feature matrix (*X*) of the graph as input. The adjacency matrix contains the linkages and their weight information between each node, while the node embedding obtained by weighted node2vec serves as the feature matrix.

## Experiments

In this section, we demonstrate the effects of the scaling algorithm on real-world datasets. Specifically, we show how the average, standard deviation, and range of ratings change when the algorithm is applied, as well as how it adjusts specific users’ rating patterns. We also compare the node embeddings obtained from the scaled version and the original version, both with 32 dimensions, through three tasks: i) classifying good and bad users, ii) link prediction, and iii) node clustering. Classifying good users and bad users is our main task because it is essential in anonymous trading networks, while the other two tasks are sub-tasks that help compare the representation power of nodes.

### Parameter setup

In this section, we provide an explanation of the hyper-parameters utilized in this paper. The explanation is divided into two main parts. In the first part, we describe the parameters employed in the scaling algorithm. In the second part, we elucidate the parameters utilized in the embedding module. First, Table 1 illustrates the distribution of ratings in the datasets derived by varying the values of *w* in Eq 2 and *α* in Fig 3, among the hyper-parameters used in the scaling algorithm. The period corresponding to *α* was set considering the duration of dataset collection, ranging from 30 days (one month) to 720 days (approximately 2 years). The value of *w* was set to $\frac{1}{8}$, $\frac{1}{4}$, $\frac{1}{2}$, and $\frac{7}{8}$ to examine the standard deviation of ratings. As a result, for the Bitcoin-Alpha dataset, the standard deviations of all combinations of *α* and *w* increased by at least 1.19 times compared to the original standard deviation. In the case of the Bitcoin-OTC dataset, it increased by at least 1.15 times, and for Wiki-RfA, it increased by at least 1.21 times. Specifically, the results generally demonstrate that as *α* increases (indicating longer unit periods) and as *w* increases, the standard deviation tends to increase. However, in cases where both *α* and *w* were increased simultaneously, there was a tendency for the standard deviation to decrease instead. We performed grid search to optimize the hyper-parameters, and the optimal values were found to be *α* of 30 and *w* of $\frac{1}{2}$.

**Table 1:** The standard deviation of ratings for each dataset according to changes in the parameters *α* (unit period) and *w* (time-decay weight).

Bitcoin-Alpha		Weights (*w*)			
		1/8	1/4	1/2	7/8
Days (*α*)	30	3.4745	3.4778	3.4864	3.5053
	60	3.4911	3.4939	3.5002	3.5103
	180	3.5066	3.5082	3.5116	3.5163
	360	3.5136	3.5144	3.5158	3.5176
	720	3.5148	3.5150	3.5153	3.5157
Original		2.9036			
Bitcoin-OTC		Weights (*w*)			
		1/8	1/4	1/2	7/8
Days (*α*)	30	4.1237	4.1319	4.1518	4.1960
	60	4.1537	4.1609	4.1767	4.2017
	180	4.1754	4.1805	4.1907	4.2038
	360	4.1903	4.1933	4.1984	4.2041
	720	4.1962	4.1968	4.1979	4.1990
Original		3.5620			
Wiki-RfA		Weights (*w*)			
		1/8	1/4	1/2	7/8
Days (*α*)	30	0.5231	0.5229	0.5229	0.5234
	60	0.5230	0.5230	0.5232	0.5238
	180	0.5231	0.5232	0.5234	0.5239
	360	0.5233	0.5234	0.5236	0.5239
	720	0.5235	0.5236	0.5237	0.5238
Original		0.4307			

Secondly, we elaborate on the hyper-parameters settings for the weighted graph embedding module. The graph embedding module encompasses weighted node2vec and GAE, as depicted in Fig 5. Firstly, for the node2vec hyper-parameters, the maximum length of a random walk was set to 10, and the number of random walks per root node was set to 10. The return parameter, denoted as *p*, was set to 0.5, while the in-out parameter, denoted as *q*, was set to 2. Additionally, the dimensionality of the node vector representation was set to 32. The representations of nodes obtained through weighted node2vec are used as features for each node and fed into the GAE. In this process, GAE comprises two GCN layers with a hidden unit count of 32. Furthermore, the learning rate was set to 0.001, the number of epochs was 200, weight decay was 0.1, and dropout was set to 0.5. These settings were referenced from configurations used in the studies of node2vec and GAE. Parameters were adjusted heuristically, iteratively seeking the optimal parameter values.

### Preliminary results for scaling effect

**Fig 6:**
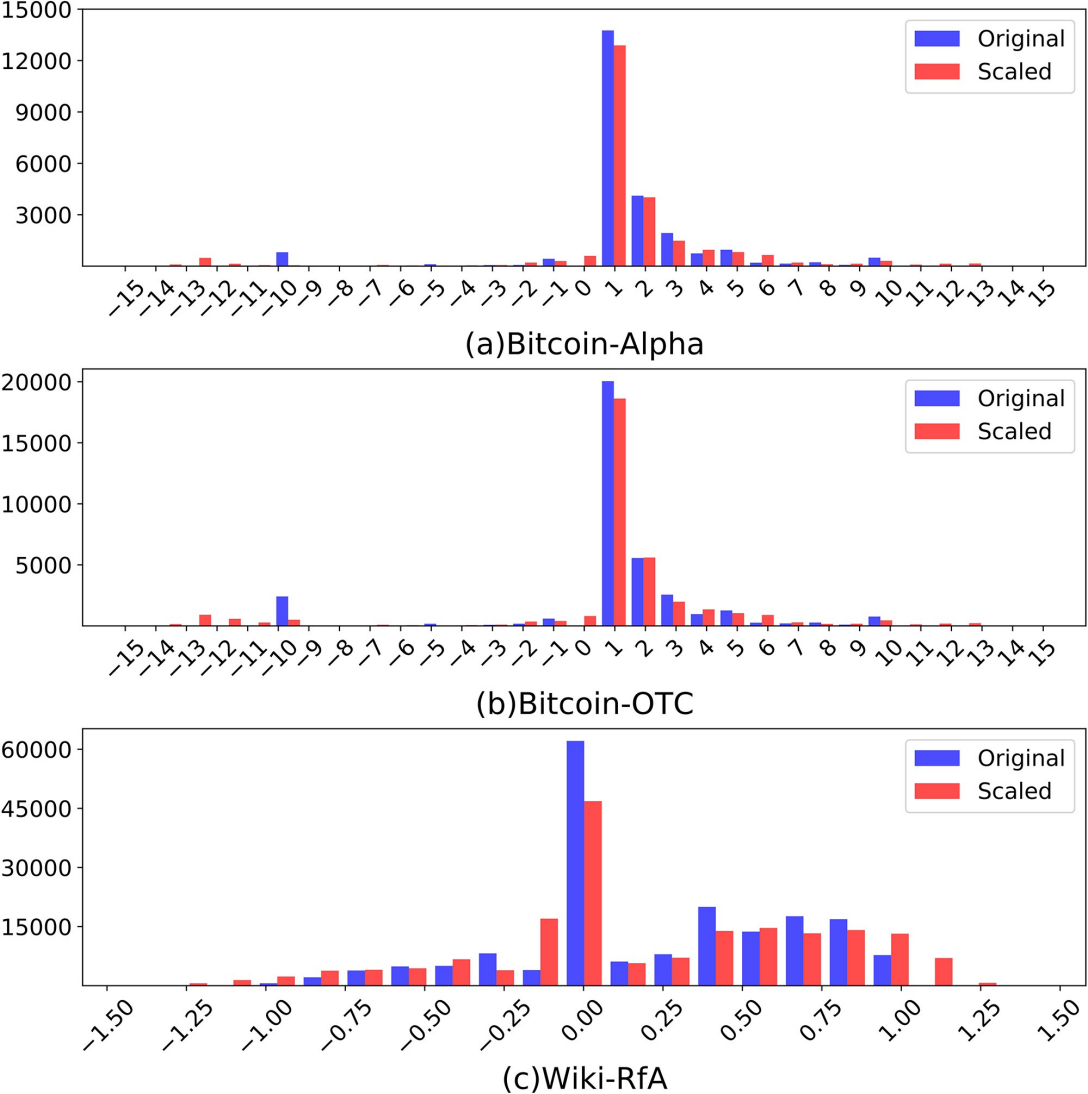
The histogram of the rating distribution before and after scaling for the three datasets. It shows the distribution of the original rating and scaled rating. The blue bar represents the number of original ratings, and the red bar represents the number of scaled ratings, which is 1.5 times larger than the original rating range. (a) Bitcoin-Alpha. (b) Bitcoin-OTC. (c) Wiki-RfA.

As shown in Fig 6, by setting *θ* in Eq 1 to 4 for all three datasets, the range of the scaled rating for each dataset was increased by a factor of 1.5. The hyper-parameter *θ* is a value heuristically set, where a smaller value is set to give more weight to past ratings, and a larger value is set to give more weight to current ratings. Concentration in the central part (1 point for the two Bitcoin datasets and 0 points for the Wiki-RfA dataset) can be shown in both the original rating and the scaled rating, but the scaled rating has a slightly more spread distribution. Some of the quantified properties are described in Table 2 below.

**Table 2:** Dataset properties before and after applying the scaling algorithm.

Dataset	Bitcoin-Alpha		Bitcoin-OTC		Wiki-RfA	
Scaling	Before	After	Before	After	Before	After
# of transactions	24,168		35,592		180,864	
Range of rating	[-10,+10] except 0	[-15,+15] except 0	[-10,+10] except 0	[-15,+15] except 0	[-1,+1]	[-1.5,+1.5]
Average	1.4639	1.4518	1.0120	0.9731	0.2208	0.2185

Table 2 shows the dataset properties before and after applying the scaling algorithm. In this case, for the hyper-parameters, as mentioned in Section 1, *α* was set to 30, and *w* was set to $\frac{1}{2}$. Considering that the two Bitcoin datasets don’t have a 0 point and the default value is 1, the average value is close to 1 before applying the scaling algorithm. In the case of the Wiki-RfA dataset defined based on sentiment analysis of text, the original average is 0.2208. What we should note is that when the scaling algorithm is applied to all three datasets, as shown in Table 1, the standard deviation increases, and, as shown in Table 2, the average decreases. In other words, an increase in the standard deviation means that the ratings concentrated in a narrow range are spread out due to the users’ tendency to give ratings. In addition, the decrease in the average rating means that there are many cases in which the evaluation of users with bad reputations is maximized by the scaling algorithm more than users with good reputations.

**Fig 7:**
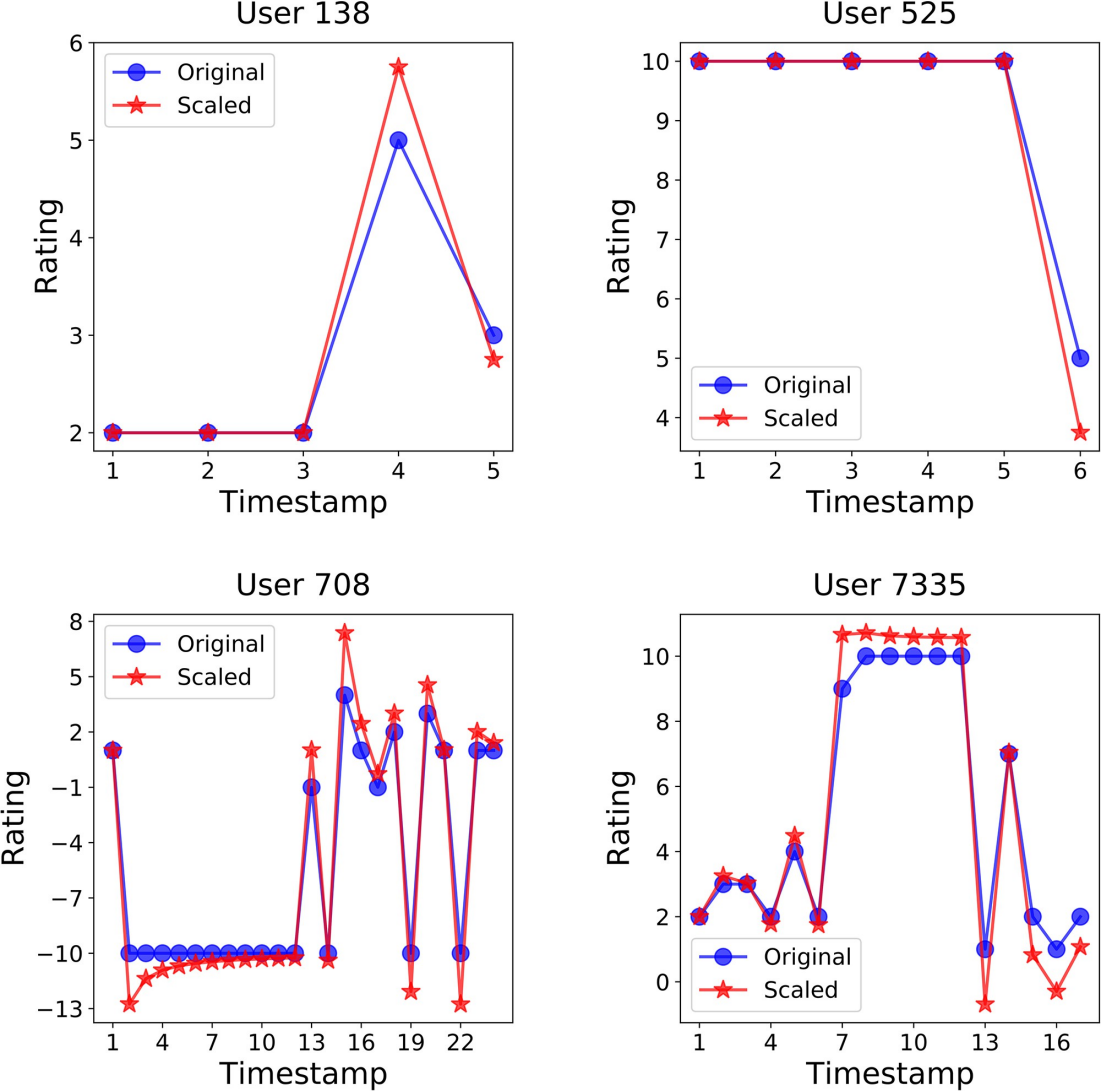
Record of ratings given by four users in the Bitcoin-Alpha dataset. It shows the rating history of four anonymous users. The blue line represents the original rating given by the user, and the red line represents the adjusted rating by the scaling algorithm.

We observed four specific users in the Bitcoin-Alpha dataset as shown in Fig 7. Two of them had a relatively low number of transactions and a simple giving rating pattern, while the other two had a relatively high number of transactions and a more complex pattern. For example, user 138, considering timestamps 1 to 3, tended to give + 2 points if satisfaction with the transaction was common, but gave + 5 points at timestamp 4 when the satisfaction was better than before. This value was adjusted to +5.75 points, which is higher than +5 to match the goal of this work. As another example, the rating that user 525 habitually gave on timestamps 1 to 5 is +10. However, user 525 was more dissatisfied with the transaction at timestamp 6 than before, giving +5 instead of the usual +10. This +5 point was adjusted to +3.75 by the proposed algorithm, reflecting the intention of *unsatisfactory*. Likewise, user 708 tended to give −10 points in normal times (timestamps 2 through 12). The user 708 gave −1 point to the transaction at timestamp 13. −1 point, of course, means bad with a negative value, but compared to the −10 points given 11 times before, it represents a large value. After that, user 708 gave −10 points at timestamp 14, and gave a positive value of +4 points at timestamp 15. Considering the tendency to award −10 points several times in the past, the +4 points were adjusted to +7.37 points with the addition of *satisfactory*. Finally, user 7335 gave ratings between +2 and +4 for timestamps 1 through 6. The +9 points given by user 7335 at Timestamp 7 were adjusted to +10.66 points based on the history of previous points.

As such, we focused on the ratings that users have given in the past based on *habits or inclinations*, and reflect them in the current rating. As demonstrated in this section, this approach effectively achieved the goal of our study. We performed embedding learning with graph-structured data, including adjusted ratings, and conducted experiments on three tasks that compared learned embeddings using original ratings.

### Classification for good users and bad users

We referred to the settings used by S. Kumar et al. [1, 2] to define good and bad users. In their studies, the scale of all ratings was adjusted to [−1, +1], and a user with a rating of −0.5 or less was considered highly negative, while a user with a rating of + 0.5 or more was considered highly positive. Building on their definition, we defined users with an average of original ratings of −5 or less as bad users and users with an average of original ratings of + 5 or more as good users, taking into account the original rating range of [−10, +10] in the Bitcoin-Alpha and Bitcoin-OTC datasets. Classified by these criteria, Bitcoin-Alpha has 144 good users and 108 bad users, and Bitcoin-OTC has 168 good users and 345 bad users. Similarly, since the rating range of the Wiki-RfA dataset defined based on text sentiment analysis is [−1, +1], users with an average of original ratings of −0.5 or less were defined as bad users, and users with an average of original ratings of +0.5 or more were defined as good users. However, dividing by these criteria, there are only 55 good users and only 6 bad users. Therefore, only for Wiki-RfA, users with an average rating of +0.5 or higher were defined as good users, and users with an average rating of −0.2 or lower were defined as bad users, resulting in 55 good users and 40 bad users. We call the classification of users by the aforementioned criteria *classification for dramatic users*.

Additionally, we performed a second classification that includes all users. For all datasets, users with a positive average rating value were classified as good users, and those with a negative average rating value were classified as bad users. Using this criterion, Bitcoin-Alpha had 3,354 good users and 400 bad users, Bitcoin-OTC had 4,857 good users and 1,001 bad users, and Wiki-RfA had 3,156 good users and 312 bad users. We refer to this classification as the *classification for all users*.

We used a total of five classifier models, Stochastic Gradient Descent (SGD)-based Linear classifier model (Linear) [28], Decision Tree (DT) [29], Support Vector Machine (SVM) [30], Random Forest (RF) [31], and Multi-Layer-Perceptron (MLP) [32, 33]. The MLP model for binary classification had three fully connected layers, each with 32 units, and ReLU as the activation function. The output layer had two units, the number of classes, and a sigmoid activation function. We used the default hyper-parameters for the remaining four classification models, which were provided by the python scikit-learn library [34].

Table 3 shows the accuracy of the embeddings obtained using the original ratings and the embeddings obtained using the scaled ratings, applied to the five classifier models including MLP. By applying MLP, in the case of Bitcoin-Alpha, it was learned while adjusting the train ratio to 80%, 60%, and 40% for 144 good users and 108 bad users, and shows the accuracy for each test data. This demonstrates that the classification performance of the scaled version outperforms that of the original version for all train ratios by 15% to 17%, which is the greatest performance improvement compared to the other datasets. In the classification problem for Bitcoin-OTC, which consists of 168 good users and 345 bad users, the scaled version outperforms the original version, with an improvement of 3% to 5% for all train ratios. Finally, in the Wiki-RfA dataset, the original version performs slightly better when the train ratio is 40%, and the scaled version outperforms it, with and improvement of 5% to 9%, when the train ratio is 60% and 80%. However, considering that Wiki-RfA consists of 55 good users and 40 bad users, the number of data corresponding to a train ratio of 40% is less than 40, which means a relatively small number of training data.

**Table 3:** Accuracy of classification for dramatic users using original rating and scaled rating by utilizing classifiers.

Classifier Methods	Dataset	Version	Accuracy (Train ratio)		
			80%	60%	40%
SGD	Bitcoin-Alpha	Original	0.7451	0.7327	0.7303
		Scaled	**0.9020**	**0.9208**	**0.9408**
	Bitcoin-OTC	Original	**0.7864**	0.7621	0.7500
		Scaled	0.7767	**0.8058**	**0.7792**
	Wiki-RfA	Original	0.4737	**0.6053**	0.5614
		Scaled	**0.7368**	0.5063	**0.6667**
DT	Bitcoin-Alpha	Original	0.6667	0.6040	0.6974
		Scaled	**0.8627**	**0.9406**	**0.8947**
	Bitcoin-OTC	Original	0.7767	0.7816	0.7468
		Scaled	**0.8058**	**0.7864**	**0.7857**
	Wiki-RfA	Original	0.6316	0.6316	0.5439
		Scaled	**0.6842**	**0.6842**	**0.5614**
SVM	Bitcoin-Alpha	Original	0.7843	0.7426	0.7566
		Scaled	**0.9216**	**0.9208**	**0.9145**
	Bitcoin-OTC	Original	0.7864	0.7961	0.8052
		Scaled	**0.8252**	**0.8592**	**0.8247**
	Wiki-RfA	Original	0.6842	0.6579	0.4561
		Scaled	**0.7895**	**0.7105**	**0.4912**
RF	Bitcoin-Alpha	Original	0.8235	0.7921	0.7632
		Scaled	**0.9216**	**0.9307**	**0.9276**
	Bitcoin-OTC	Original	0.8155	0.8252	0.8052
		Scaled	**0.8835**	**0.8883**	**0.8442**
	Wiki-RfA	Original	0.6316	0.6316	0.5614
		Scaled	**0.6842**	**0.7632**	**0.6316**
MLP	Bitcoin-Alpha	Original	0.7843	0.7624	0.7237
		Scaled	**0.9412**	**0.9307**	**0.9211**
	Bitcoin-OTC	Original	0.8350	0.8204	0.8177
		Scaled	**0.8835**	**0.8574**	**0.8409**
	Wiki-RfA	Original	0.6842	0.6316	**0.5789**
		Scaled	**0.7368**	**0.7242**	0.5614

For all other classifier models except MLP, in the case of the Bitcoin-Alpha dataset, the classification accuracy of the original version and the scaled version showed the highest performance in the RF classifier among the four classifiers. The accuracy of the original version was 0.8235 (train ratio 80%), 0.7921 (60%), and 0.7632 (40%), which are higher than the MLP classifier. However, the scaled version showed higher accuracy of 0.9216 (train ratio 80%), 0.9307 (60%), and 0.9276 (40%), and for all classifier models including RF, the scaled version showed higher accuracy than the original version. Likewise, the Bitcoin-OTC dataset also showed the best performance when the RF classifier was applied than the other three classifiers (DT, SVM, and SGD). When classified by applying the original version, DT, SVM, and SGD shows an accuracy of 0.7468 to 0.8052, but when RF was applied, an accuracy of up to 0.8252 was shown. What we should note here is that the scaled version shows better classification accuracy than the original version for all train ratios of all classifiers, except for the part where the train ratio of the SGD classifier model is 80%. In the case of the Wiki-RfA dataset, the classification accuracy is lower than the two Bitcoin datasets because the number of training data is significantly smaller than that of the two Bitcoin datasets, but the scaled version still shows higher accuracy than the original version.

The classification performance shown earlier was when the classes were defined as very bad users and very good users. The results shown this time are the performance of classifying users with a positive average reputation as good users and users with a negative average reputation as bad users for each dataset. That is, all users must belong to one of the two classes. The Bitcoin-Alpha dataset has about 11% negative class, Bitcoin-OTC has about 17%, and Wiki-RfA has only about 9%. We faced a class imbalance issue and measured performance using the balanced accuracy metric, defined as the average of sensitivity ($=\frac{\text{TP}}{\text{TP}+\text{FN}}$) and specificity ($=\frac{\text{TN}}{\text{TN}+\text{FP}}$). This metric allows for a comprehensive evaluation by considering both the true positive rate and the true negative rate. The F1-score metric, which is the harmonic mean of precision ($=\frac{\text{TP}}{\text{TP}+\text{FP}}$) and sensitivity ($=\frac{\text{TP}}{\text{TP}+\text{FN}}$), tends to emphasize the performance of the positive class. Therefore, it can be biased towards the positive class in imbalanced datasets. However, the balanced accuracy metric can reflect the performance for both the positive and negative classes in a balanced manner. Hence, we adopted the balanced accuracy suitable for the class distributions in our datasets [35, 36]. In addition, we additionally used loss that varies the weight according to the number of data in the class to the loss function in MLP.

Table 4 shows the results of classification by applying the original version and the scaled version to the five classifiers including MLP for all users in each dataset. By applying MLP, even when the classification was performed for all users, the scaled version showed the greatest performance improvement in the Bitcoin-Alpha dataset. Compared to the original version, the scaled version showed an improvement between 11% and 13% for all train ratios and showed the highest balanced accuracy when the train ratio was 60%. In the case of the Bitcoin-OTC dataset, the scaled version showed higher accuracy than the original version for all train ratios. On the other hand, the Wiki-RfA dataset showed almost similar performance to the original version and the scaled version and did not show the effect of scaling. Wiki-RfA dataset has the most severe class imbalance, and unlike two Bitcoin datasets, it has not seen a good scaling effect in that it uses ratings via text-based emotional analysis, rather than ratings given directly by users.

**Table 4:** Balanced accuracy of classification for all users using original rating and scaled rating by utilizing classifiers.

Classifier Methods	Dataset	Version	Balanced Accuracy (Train ratio)		
			80%	60%	40%
SGD	Bitcoin-Alpha	Original	-	-	-
		Scaled	-	-	-
	Bitcoin-OTC	Original	0.5275	0.5355	0.5349
		Scaled	**0.6333**	**0.5779**	**0.5933**
	Wiki-RfA	Original	-	-	-
		Scaled	-	-	-
DT	Bitcoin-Alpha	Original	0.6246	0.5669	0.5922
		Scaled	**0.7013**	**0.6884**	**0.6828**
	Bitcoin-OTC	Original	0.6534	0.6688	0.6630
		Scaled	**0.6904**	**0.6984**	**0.6980**
	Wiki-RfA	Original	0.5500	0.5551	0.5507
		Scaled	**0.5914**	**0.5784**	**0.5680**
SVM	Bitcoin-Alpha	Original	0.4992	-	-
		Scaled	**0.6178**	**0.6008**	**0.5627**
	Bitcoin-OTC	Original	0.5697	0.5663	0.5533
		Scaled	**0.6402**	**0.6304**	**0.6267**
	Wiki-RfA	Original	-	-	-
		Scaled	-	-	-
RF	Bitcoin-Alpha	Original	0.5866	0.5807	0.5630
		Scaled	**0.7310**	**0.7116**	**0.7023**
	Bitcoin-OTC	Original	0.6862	0.6632	0.6523
		Scaled	**0.7079**	**0.7169**	**0.7014**
	Wiki-RfA	Original	0.5145	**0.5302**	0.5434
		Scaled	**0.5343**	0.5263	**0.5436**
MLP	Bitcoin-Alpha	Original	0.6390	0.6324	0.6107
		Scaled	**0.7492**	**0.7596**	**0.7434**
	Bitcoin-OTC	Original	0.6877	0.7085	0.7004
		Scaled	**0.7384**	**0.7368**	**0.7335**
	Wiki-RfA	Original	0.5144	**0.5417**	**0.5336**
		Scaled	**0.5216**	0.5313	0.5321

For all other classifier models except MLP, the RF classifier showed the highest performance for the two Bitcoin datasets, as in the case of classifying dramatic users. Applying the original and scaled versions of the Bitcoin-Alpha dataset to RF resulted in 13% to 15% performance improvement, and for the Bitcoin-OTC dataset, the improvement was 3% to 5%. However, when the Bitcoin-Alpha dataset was applied to the Linear classifier, the classification performance of both the original and scaled versions was as low as 0.5, which means the classifier classified all data into one class in binary classification (marked as “-” in the table). For the Bitcoin-OTC dataset, the scaled version outperformed the original version for all four classifier models. In the Wiki-RfA dataset, among the five classifier models, including the MLP classifier, DT showed excellent performance in both the original and scaled versions. However, the SVM and Linear classifiers failed to classify good users and bad users in both the original and scaled versions of Wiki-RfA.

As a result, the scaling method proved to be effective for the two Bitcoin datasets that contained ratings based on numbers directly assigned by users, resulting in significant improvements in classification performance, especially in the Bitcoin-Alpha dataset. To demonstrate this performance improvement, we conducted a classification experiment by changing the criteria for distinguishing good users from bad users. By applying the scaling method, it became possible to treat good users better and bad users worse, which was verified through quantitative evaluation using accuracy and balanced accuracy metrics. Comprehensively, in the case of classification for dramatic users, out of a total of 45 cases (combination of 3 datasets, 5 classifiers, and 3 train ratios), the scaled version showed better performance in 42 cases. Similarly, as a result of the experiment for classification for all users, the scaled version outperformed the original version in 33 cases out of a total of 36 cases, except for the case where the classifier with a balanced accuracy of 0.5 did not work properly.

**Fig 8:**
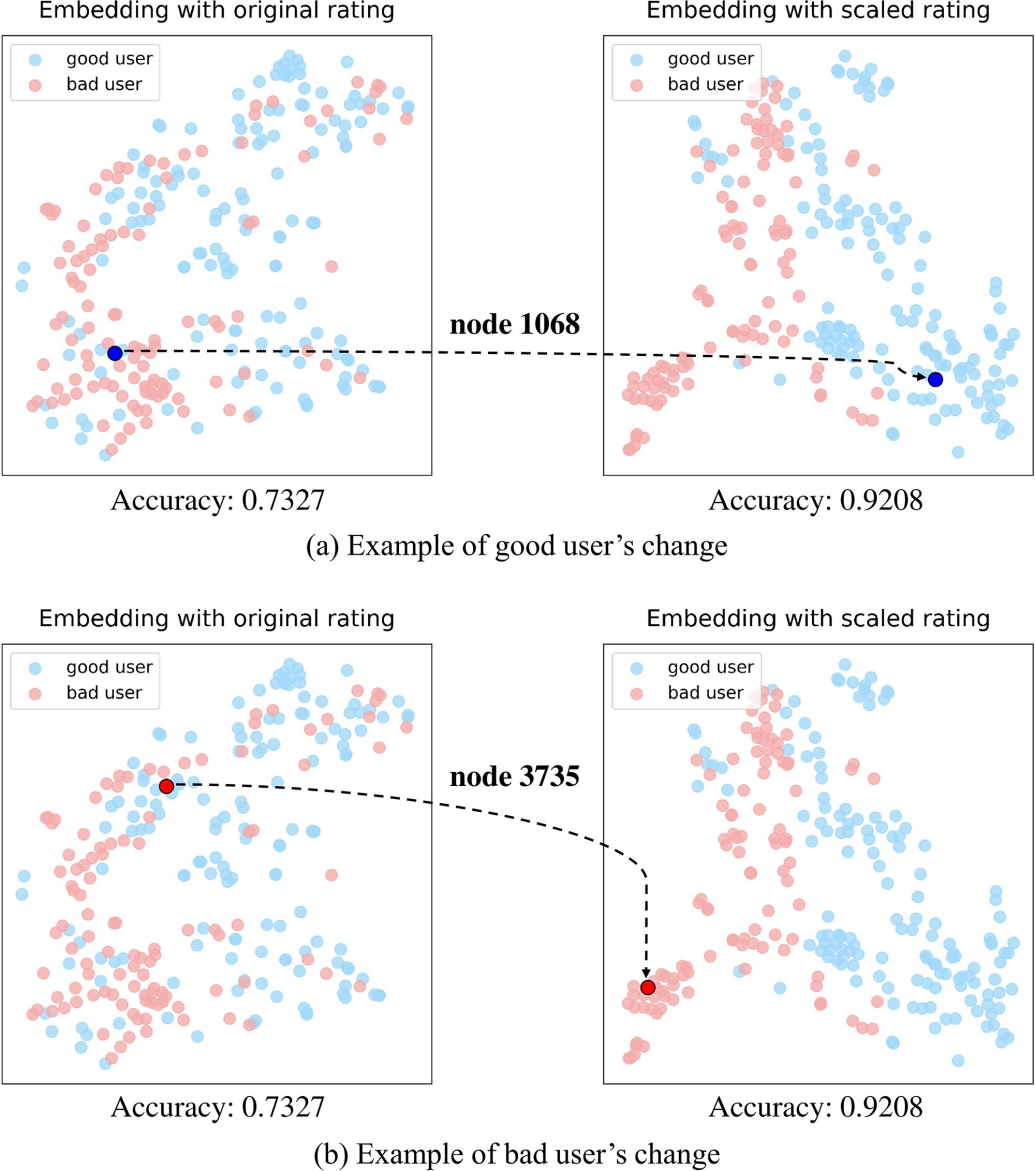
Embedding plot reflecting original rating and scaled rating of Bitcoin-Alpha dataset. Users with an average rating of + 5 points or more are defined as good users, and users with an average rating of −5 points or less are defined as bad users. Green dots represent good users, yellow dots represent bad users. (a) Example of good user’s change. (b) Example of bad user’s change.

In addition to quantitative analysis, we also conducted a qualitative analysis. As shown in Fig 8, the dramatic users (Users with an average reputation of + 5 or higher are good users, and users with an average reputation of −5 or lower are bad users) of the Bitcoin-Alpha dataset are divided into two classes, and the results of embedding results with different colors are shown. In both the left and right figures of (a) and (b) in Fig 8, blue dots represent good users, while red dots represent bad users. In both Fig 8(a) and 8(b), the left side is the result of applying the original rating to our embedding module, and the right side is the result of applying the scaled rating to the embedding module. In the embedding using the original version (left), there are quite a few parts where good users and bad users are mixed. In other words, embedding using original rating information could not reflect the difference between good users and bad users relatively well. In contrast, the embedding reflecting the scaled rating (right) is expressed so that good users and bad users can be distinguished well. In particular, the blue dot in Fig 8(a) represents node 1068 with an average received rating of + 7 points. In the embedding using the original ratings on the left, it is positioned among the bad users, but in the embedding using the scaled ratings on the right, it resides in proximity to the good users. Likewise, the red dot in Fig 8(b) represents node 3735 with an average received rating of −6.5 points. Prior to scaling, it was positioned among the good users, but after scaling, it has been relocated to where the bad users gather.

We utilized an embedding module that combined weighted node2vec [5] and GAE [6] to perform node embedding. The original and scaled ratings were assigned as the edge weight between two users to capture the difference between good and bad users, which was taken into account while performing weighted node2vec and GAE to obtain explicit representations of each user. Due to the characteristics of weighted node2vec, the probability of extending to other nodes when performing a random walk varies according to the edge weight. By utilizing the scaling method, we increased the variance of ratings, allowing good users to be rated better and bad users to be rated worse. Thus, when the weighted node2vec is conducted, these points are taken into account to obtain an explicit representation of good and bad users. Using this explicit representation as an feature of each node, we applied GAE, a GNN-based embedding model, and performed final node embedding learning by collecting information on neighbor users of each user. By performing a task to classify good and bad users using the final two versions of embeddings, we found that embedding representations using scaled rating better classify good and bad users than embedding representations using the original rating for a total of five classifier models including MLP classifiers.

### Link prediction

Link prediction in a complex network is the task of discovering where there is a tentative relationship between two different nodes, but in reality, there is no linkage. Link prediction is a task used in applications such as friend or user recommendations and item recommendations in social networks and e-commerce. In addition, studies on graph embedding techniques that transform into a low-dimensional vector space while preserving the relationship information of nodes in the network as much as possible perform a connection prediction task to verify the representation power of the node embedding projected into the vector space. The graph embedding module used in this study consists of weighted node2vec and GAE.

We conducted an experiment to verify which version of the embedding obtained from the node embedding with the original rating and the node embedding with the scaled rating had more explicit node representations. This task is performed when graph reconstruction is conducted by decoding the embedding during the embedding learning process through the GAE model. To conduct the link prediction experiment, we generated negative samples for node pairs that do not actually exist, in addition to the positive samples present in the real dataset. Subsequently, the positive and negative samples were divided into the train set (70%), validation set (20%), and test set (10%) in equal proportions for learning, and the inference performance was measured. The source code of GAE was publicly provided by the author, and the hyper-parameters and other experimental settings were not modified.

We measured link prediction performance, the first sub-task, to verify the effect of the scaling method. Table 5 shows a comparison of the performance of the original version and the scaled version. Before discussing performance, looking at the four metrics, True Positive (TP) means that a link actually exists between two nodes and the model predicts that the link will exist. True Negative (TN) means that the link between the two nodes does not actually exist, and the model also predicts that the link does not exist. False Negative (FN) is when a link actually exists, but the model predicts that the link does not exist. Finally, False Positive (FP) means that the link does not actually exist, but the model predicts that the link will exist. AUC ROC [37] is a binary classification performance metric calculated using the True Positive Rate (TPR) and False Positive Rate (FPR), and has a value between 0 and 1, and closer to 1 means better performance.

$$\begin{array}{c} \begin{array}{c} Precision=\frac{TP}{TP+FP} \end{array} \end{array}$$ (5)

$$\begin{array}{c} \begin{array}{c} Recall=\frac{TP}{TP+FN} \end{array} \end{array}$$ (6)

$$\begin{array}{c} \begin{array}{c} F1\;score=\frac{2}{\frac{1}{Precision}+\frac{1}{Recall}} \end{array} \end{array}$$ (7)

**Table 5:** Link prediction performance using original version and scaled version.

Dataset	Version	AUC ROC	Precision	Recall	F1-score
Bitcoin-Alpha	Original	0.9190	0.6330	0.9608	0.7631
	Scaled	**0.9395**	**0.6461**	**0.9734**	**0.7767**
Bitcoin-OTC	Original	0.9223	0.6275	0.9672	0.7611
	Scaled	**0.9462**	**0.6473**	**0.9740**	**0.7777**
Wiki-RfA	Original	0.9753	0.5876	0.9960	0.7391
	Scaled	**0.9781**	**0.6028**	**0.9968**	**0.7512**

Precision and recall are calculated by Eqs 5 and 6, respectively, and precision means the ratio of those that actually have a connection that the model predicts that there is a connection. Recall means that among those predicted by the model that there will be a connection, there is actually a connection. The F1 score [38] is calculated as the harmonic average of precision and recall as in Eq 7.

As shown in Table 5, representing the comprehensive results for link prediction, both the original version and the scaled version show significantly higher recall than precision. This is considered to be the result of what happened, regardless of the difference between the original rating and the scaled rating. It should be focused that the use of scaled versions for all datasets showed a slight performance improvement in the link prediction task compared to the use of original versions. This also proves that explicit node representations are obtained when learning embedding using scaled ratings rather than learning embedding using original ratings.

### Clustering

The second sub-task was clustering, an unsupervised learning method used to evaluate how dense users’ embeddings gather. We used two clustering algorithms, hierarchical agglomerative clustering (HAC) [39, 40] and K-Means [41]. HAC is a bottom-up approach that merges clusters based on the distance of the two closest data or the two most distant data in two different clusters. We used the ward linkage method [42] in HAC, which merges two clusters that increase the variance in all clusters the least. The optimal number of clusters was 3 for the two Bitcoin datasets and 4 for the Wiki-RfA dataset. K-Means clustering is based on a top-down approach and the algorithm works by the following process. First, randomly selects *k* centroids. Second, assigns each data point to a cluster based on the similarity between each data point and centroids. Third, move the center to the center of the cluster. Finally, repeat steps 2 and 3 until the centroid no longer moves. The K-means algorithm requires that the number of clusters (*k*) is predefined. We varied *k* from 2 to 10 for each dataset, and after clustering, the optimal *k* was determined based on the elbow method [43] by calculating the cohesion of the cluster by summing the distances between the cluster data at each center point. As a result, likewise HAC, the optimal number of clusters for the two Bitcoin datasets was determined to be 3 and the optimal number of clusters for Wiki-RfA was determined to be 4.

We used four metrics to measure clustering performance for clusters without ground-truth information. The first metric, the silhouette score [44], is the most commonly used index and is calculated via the intra-cluster distance and inter-cluster distance. The silhouette score has a value between −1 and +1, and the closer to +1, the better the clustering. The second metric, the Calinski-Harabasz index [45], is calculated through the variance of how far each data point is from the center of the cluster to which the data belongs, and the variance of how far each cluster is from the center of the entire dataset. This index also means that the higher the value, the clearer the clustering. The third metric, the Davis-Bouldin index [46], is calculated considering inter-cluster distance and intra-cluster variance, and a smaller value means better clustering. Finally, Dunn-index [47] is also a metric calculated by considering intra-cluster distance and inter-cluster distance such as the silhouette score, and a larger value means better clustering.

Table 6 is the performance result of clustering embeddings with the HAC algorithm, and Table 7 is the performance result of clustering with the K-means algorithm. Generally, when K-means was applied, clustering performance was higher than when HAC was applied. Specifically, compared to HAC, K-means increased the silhouette score by up to 15% (original version of the Bitcoin-OTC dataset), and the Calinski-Harabasz index increased by up to 39% (original version of the Bitcoin-OTC dataset). Likewise, Davies-Bouldin increased by up to 47% (scaled version of the Wiki-RfA dataset) and Dunn index increased by up to 30% (scaled version of the Wiki-RfA dataset). In addition to comparing the two clustering algorithms, we compared the performance of the original and scaled versions for each dataset. When the original version and the scaled version were applied to the K-means algorithm, the scaled version showed a lower Calinski-Harabasz index by about 3% for the Bitcoin-Alpha dataset. For the other three metrics, the scaled version showed higher performance. In the case of Bitcoin-OTC, the silhouette score showed almost the same performance of the original version and the scaled version, the Calinski-Harabasz index was about 3% lower than the scaled version, and the Davies-Bouldin index and Dunn index improved by 2% and 9%, respectively. In the case of Wiki-RfA, the scaled version showed high values for all metrics, and in particular, the Dunn index showed an improvement of about 38% compared to the original version. Finally, in case of applying HAC, out of a total of 12 comparisons (combinations of 3 datasets and 4 metrics), the scaled version outperformed in 8 cases, and by applying K-means, the scaled version outperformed in 9 cases.

**Table 6:** Clustering performance of each version using HAC algorithm.

Dataset	Version	Silhouette score	Calinski-Harabasz	Davies-Bouldin	Dunn index
Bitcoin-Alpha	Original	0.2384	803.0780	1.4733	0.1520
	Scaled	**0.2498**	**1008.8029**	**1.3186**	**0.1840**
Bitcoin-OTC	Original	0.2005	983.8758	**1.5953**	**0.1265**
	Scaled	**0.2295**	**1105.1965**	1.7408	0.1263
Wiki-RfA	Original	0.3914	**4006.0643**	**1.1444**	0.1932
	Scaled	**0.4268**	3963.7262	1.2717	**0.2000**

**Table 7:** Clustering performance of each version using K-means algorithm.

Dataset	Version	Silhouette score	Calinski-Harabasz	Davies-Bouldin	Dunn index
Bitcoin-Alpha	Original	0.2487	**1064.1205**	1.4286	0.1712
	Scaled	**0.2681**	1033.3729	**1.2470**	**0.1866**
Bitcoin-OTC	Original	**0.2319**	**1370.0127**	1.4538	0.1280
	Scaled	0.2306	1333.1893	**1.4251**	**0.1397**
Wiki-RfA	Original	0.3771	5286.2090	0.9461	0.1879
	Scaled	**0.4466**	**5356.0962**	**0.8618**	**0.2605**

## Discussion and conclusion

We have identified the tendency to give ratings to individual users in the rating network. We focused on that since each user has a different tendency to give ratings, even the same rating could be interpreted as a different value if the user who gave the rating is different. Therefore, based on the history of ratings given by a specific user to other users in the past, we proposed a **time-considered rating scaling method** that adjusts the rating given at the present time. This scaling method has the effect of increasing the variance of the ratings so that users with a good reputation are evaluated better and those with a bad reputation are evaluated worse. To verify this effect, we performed graph embedding learning by applying the original rating and scaled rating, respectively. Afterward, using each version of embedding, the effect of the scaling method was verified through three tasks; classification for good users and bad users, link prediction, and graph clustering. As a result, when targeting dramatic users, the classification performance of the scaled version was improved by up to 16% compared to that of the original version, and when targeting all users, it was improved by up to 13%. Also, in the link prediction task, the scaled version showed slightly higher performance than the original version. Finally, even in the case of clustering, which is unsupervised learning, as a result of a comparison using various clustering evaluation scales, the scaled version outperformed the original version in many cases. Thus, this user tendency-based rating scaling method can be applied to social networks, trading networks, reputation networks, and more. Furthermore, they can be extended to user-product networks, which are not addressed in this study. In these networks, it is possible to more explicitly grasp the goodness and badness of users or products. This is expected to be beneficial for tasks such as recommending good users or products and detecting malicious users or products for appropriate actions.
